# Supplementary figures and images for: Global characterization of gene expression in the brain of starved immature Rhodnius prolixus
Source: PLoS One. 2023 Mar 3;18(3):e0282490. doi: 10.1371/journal.pone.0282490 (PMC9983911; doi:10.1371/journal.pone.0282490)

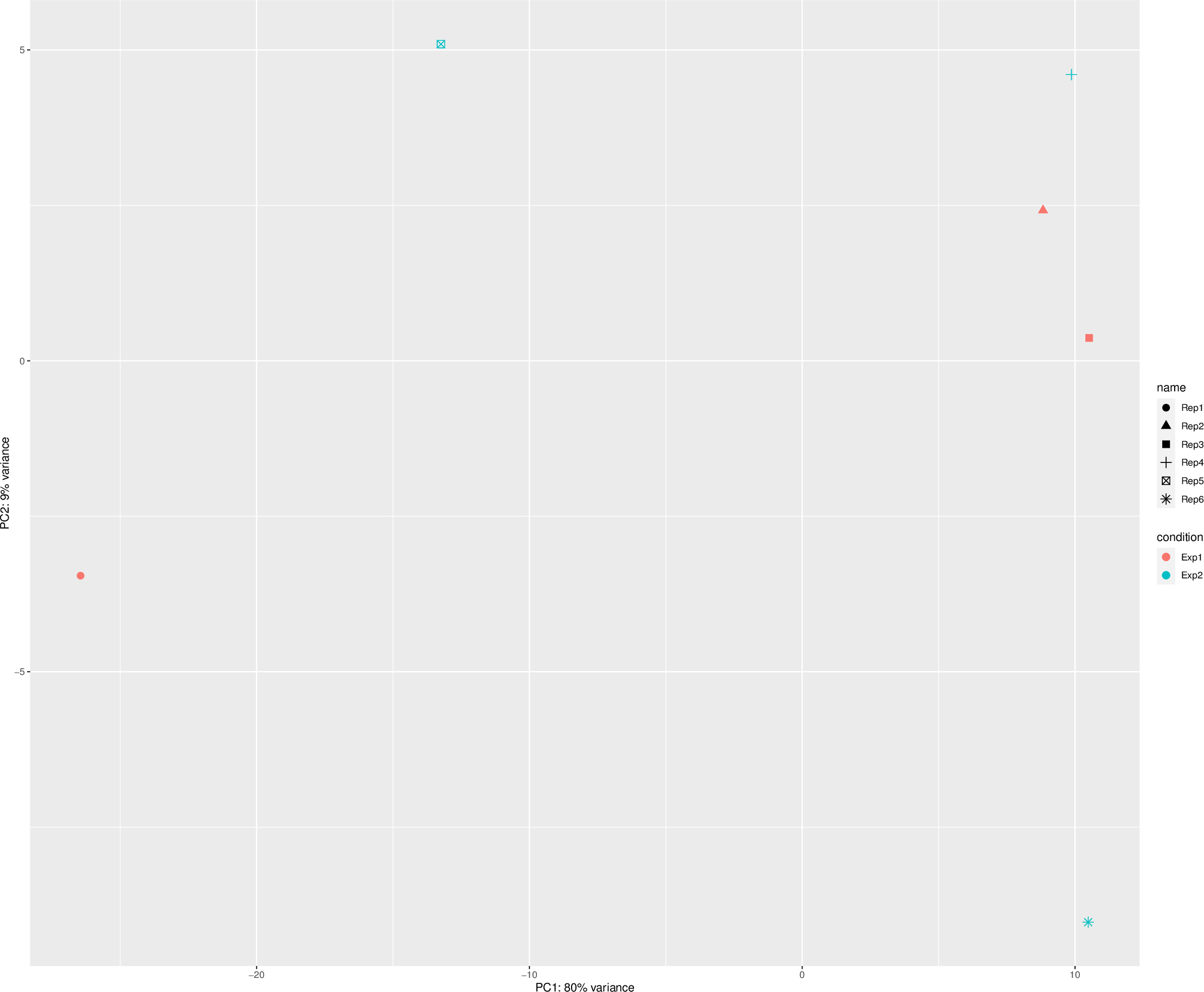

Supplement: S1 Fig — Rep 1, Rep 2, Rep 3 (experiment 1) and Rep 4, Rep 5, Rep 6 (experiment 2). (TIF) [file pone.0282490.s001.tif]

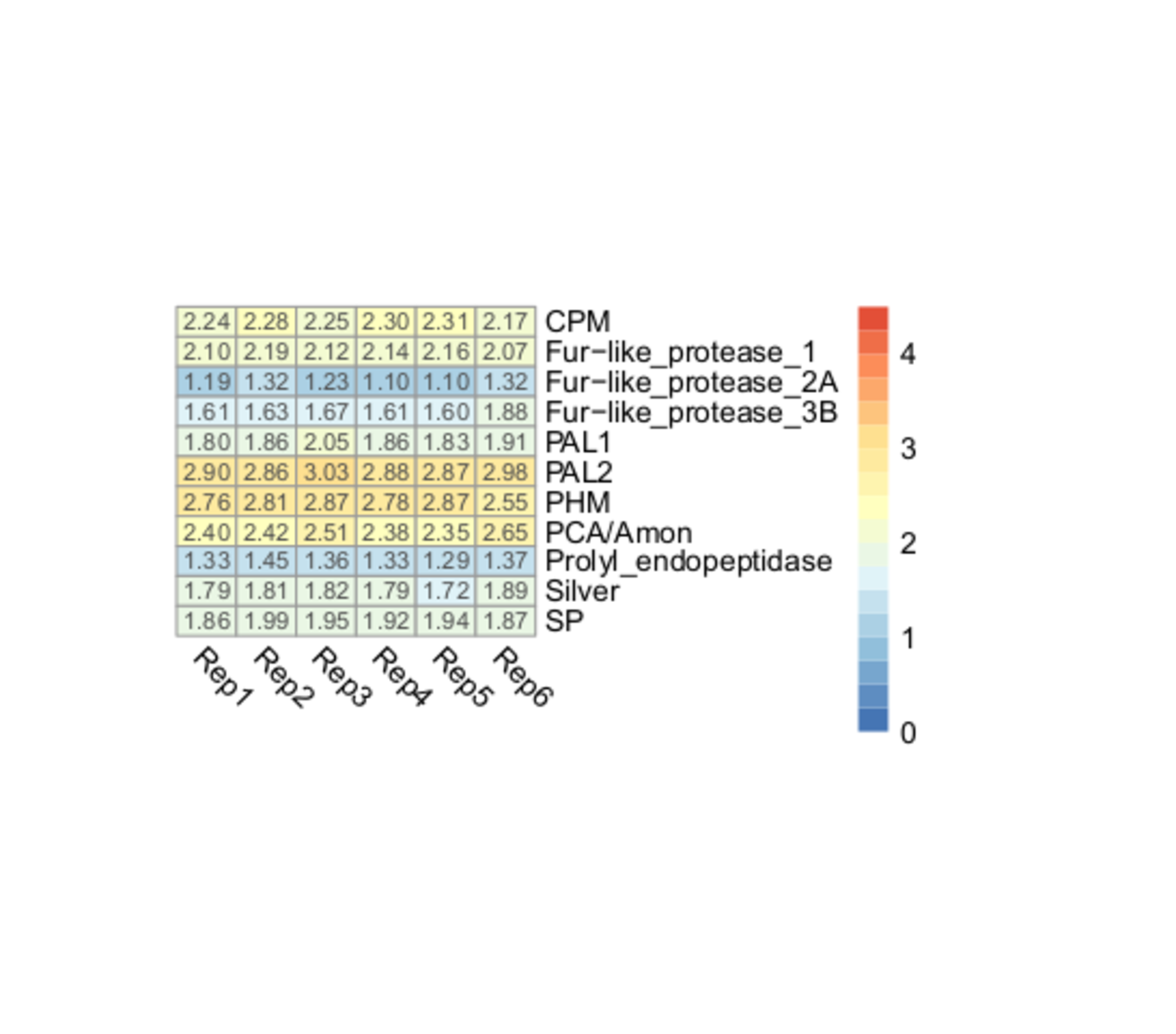

Supplement: S2 Fig — Heatmap depicting the expression level of neuropeptide processing enzymes genes in the brain of R. prolixus nymphs. Expression level (displayed as Log10 TPM+1) is represented by means of a color scale in which blue/red represent the lowest/highest expression. Each column represents the expression of one library. (TIF) [file pone.0282490.s002.tif]

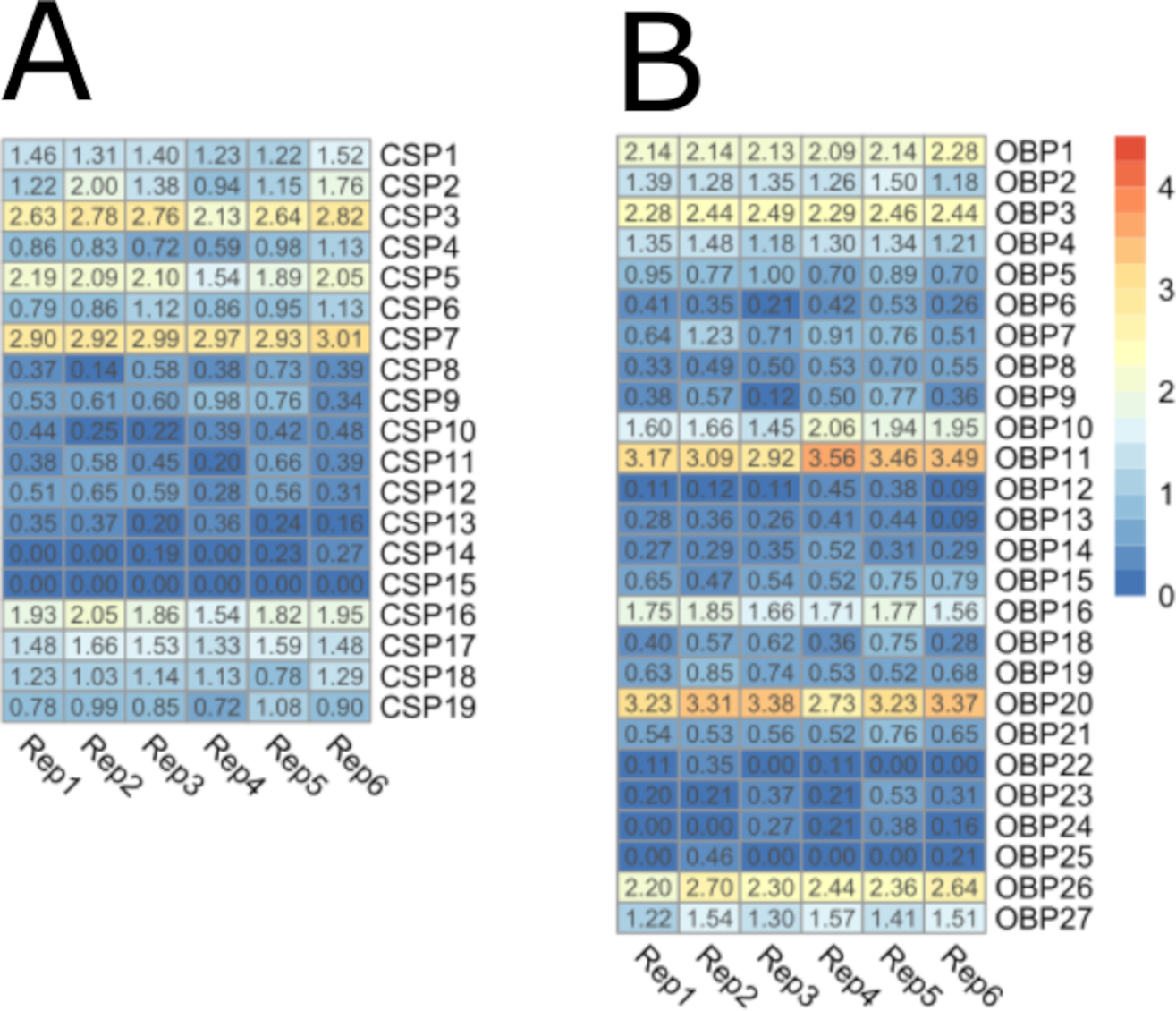

Supplement: S3 Fig — (A) Heatmap depicting the expression level of chemosensory proteins (CSPs), and (B) odorant binding protein (OBPs) genes in the brain of R. prolixus nymphs. Expression level (displayed as Log10 TPM+1) is represented by means of a color scale in which blue/red represent the lowest/highest expression. Each column represents the expression of one library. (TIF) [file pone.0282490.s003.tif]

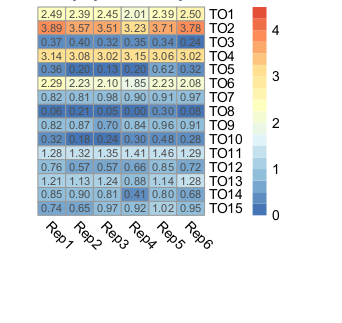

Supplement: S4 Fig — Heatmap depicting the expression level of takeout genes in the brain of R. prolixus nymphs. Expression level (displayed as Log10 TPM+1) is represented by means of a color scale in which blue/red represent the lowest/highest expression. Each column represents the expression of one library. (TIF) [file pone.0282490.s004.tif]
