# Supplementary material for: Global characterization of gene expression in the brain of starved immature Rhodnius prolixus
Source: PLoS One. 2023 Mar 3;18(3):e0282490. doi: 10.1371/journal.pone.0282490 (PMC9983911; doi:10.1371/journal.pone.0282490)
Supplement: Attachment — Submitted filename: Response_reviewers.doc [file pone.0282490.s006.doc]

Dear Dr J Joe Hull,

We are very grateful for the opportunity to respond to the reviewers’ comments and submit an improved version of our manuscript. Please, find below a point-by-point response to all comments. We hope that this new improved version can be considered for publication in PLOS ONE.

Kind regards,

Alessandra Guarneri

**In addition, I, like Reviewer 1, was a bit puzzled why fed Rhodnius were not included in the analyses. Given the reports in the literature re transcriptional regulation in response to blood feeding, it seems like directly comparing the two conditions would be more informative than one alone.**

Authors’ response: Regarding the use of six libraries of unfed bugs in our work, we want to clarify that they belong to an originally larger set of samples where the initial objective was to compare gene expression profiles between the brains of healthy and *Trypanosoma cruzi*-infected nymphs (infected either with the CL or Dm28c strains, each with 3 biological replicates*)*. We decided not to include the comparison between infected and uninfected insects in this manuscript because the differential expression analyses showed no significant differences associated with parasite infection. *Trypanosoma cruzi* is a parasite that lives exclusively in the insect’s intestinal tract, and the changes in gene expression triggered in the *R. prolixus* brain may be subtle or restricted to few neurons in specific brain regions, being masked when the whole brain is analyzed. In fact, it has been shown that even the comparison between fed and unfed *R. prolixus* adults using RNA-Seq showed no significant changes in the expression of neuropeptide and neurohormonal signaling genes in the central nervous system (Leyria et al. 2020), reflecting the challenge of detecting gene expression differences in this tissue. Therefore, we decided to use the control samples (6 replicates) to describe the overall expression of genes in unfed nymphs because we felt that describing the expression of genes potentially involved in modulating behavior during starvation will contribute to a better understanding of the functional genomics of triatomines. Nevertheless, if the editor and reviewers consider it relevant to include the data referring to *T. cruzi*, we can prepare a new version of our manuscript containing the brain libraries of *T. cruzi*-infected nymphs.

Leyria J, Orchard I, Lange AB. Transcriptomic analysis of regulatory pathways involved in female reproductive physiology of *Rhodnius prolixus* under different nutritional states. Sci Rep. 2020;10(1).

**I was also a bit surprised that AKH and sNPF transcript abundance was not higher. Could this be an artifact of the methodology or do you think it is real?**

Authors’ response: Following the editor’s comment, we have checked AKH and sNPF expression values in our databases and confirmed that both neuropeptides are expressed, presenting 835 and 918 raw counts; and 224.36 and 432.2 TPM, respectively (data are shown in S1_table). Considering that transcriptomes based on RNA samples of whole tissues are quite complex (cells are clearly not the same in expression profiles), it seems evident that these values should always be taken with a degree of caution before assuming the whole brain expresses these two genes with low expression. Indeed, it is known that the expression of many neuropeptide genes is restricted to groups of neurons. Therefore, a definitive answer will require new experiments tracking the presence of AKH and sNPF transcripts, e.g., in situ hybridization. Coincidently, RhoprSNPF peptides were detected in brain and hemolymph extracts by MS/MS (Ons et al., 2011), their presence in the CNS suggesting their local production, while hemolymph detection suggests a hormonal role (Ons, 2017).

Ons S. Neuropeptides in the regulation of *Rhodnius prolixus* physiology. J Insect Physiol. 2017;97: 77–92.

Ons S, Sterkel M, Diambra L, Urlaub H, Rivera-Pomar R. Neuropeptide precursor gene discovery in the Chagas disease vector *Rhodnius prolixus*. Insect Mol Biol. 2011;20(1):29–44.

**Line 152 should be changed to "around 13 million" rather "at least 13 million" as this is not supported by the data shown in Table 1 (ie rep 1 actually had less than 13 million reads).**

Authors’ response: We replaced “at least” by “around”, as requested.

**Table 1 shows that 67-74% of the reads across the libraries were uniquely mapped. How does this value compare with other studies?**

Authors’ response: The unique mapped read values obtained in our work are consistent with those obtained in other RNA-Seq studies performed in *R. prolixus* with the same methodology:

- Leyria, J., Orchard, I., & Lange, A. B. (2020). Transcriptomic analysis of regulatory pathways involved in female reproductive physiology of *Rhodnius prolixus* under different nutritional states. Scientific reports, 10(1), 1-16.

- Latorre-Estivalis, J. M., Große-Wilde, E., da Rocha Fernandes, G., Hansson, B. S., & Lorenzo, M. G. (2022). Changes in antennal gene expression underlying sensory system maturation in *Rhodnius prolixus*. Insect Biochemistry and Molecular Biology, 140, 103704.

- Coelho, V.L., de Brito, T.F., de Abreu Brito, I.A. et al. (2021). Analysis of ovarian transcriptomes reveals thousands of novel genes in the insect vector *Rhodnius prolixus*. Scientific Reports, 11, 1918.

**Reviewer #1:**

**However, I have a few issues with the study. For instance, I do not understand why 6 replicates of starved nymphs were analyzed, instead of comparing fed versus starved animals. This would have been more informative about the gene expression profiles and how starving actually affects gene expression in the brain.**

Authors’ response: We thank the reviewer for the interesting comments. Regarding the use of six libraries of unfed bugs in our work, we want to clarify that they belong to an originally larger set of samples where the initial objective was to compare gene expression profiles between the brains of healthy and Trypanosoma cruzi-infected nymphs (infected either with the CL or Dm28c *T. cruzi* strains, each with 3 biological replicates*)*. We decided not to include the comparison between infected and uninfected insects in this manuscript because the differential expression analyses showed no significant differences associated with parasite infection. *Trypanosoma cruzi* is a parasite that lives exclusively in the insect’s intestinal tract, and the changes in gene expression triggered in the *R. prolixus* brain may be subtle or restricted to few neurons in specific brain regions, being masked when the whole brain is analyzed. In fact, it has been shown that even the comparison between fed and unfed *R. prolixus* adults using RNA-Seq showed no significant changes in the expression of neuropeptide and neurohormonal signaling genes in the central nervous system (Leryia et al. 2020), reflecting the challenge of detecting gene expression differences in this tissue. Therefore, we decided to use the control samples (6 replicates) to describe the overall expression of genes in unfed nymphs because we felt that describing the expression of genes potentially involved in modulating behavior during starvation will contribute to a better understanding of the functional genomics of triatomines. Nevertheless, if the reviewer considers it relevant to include the data referring to *T. cruzi*, we can prepare a new version of our manuscript containing the brain libraries of *T. cruzi*-infected nymphs.

Leyria J, Orchard I, Lange AB. Transcriptomic analysis of regulatory pathways involved in female reproductive physiology of *Rhodnius prolixus* under different nutritional states. Sci Rep. 2020;10(1).

**Also, the choice of the stage is unclear to me. Why fifth instar nymphs instead of adults?**

Authors’ response: We used 5^th^ instar nymphs because we were interested in knowing the expression of genes involved in the modulation of behavior related to the search for blood sources, with no interference of those changes related to the search for a sexual partner (behavior that is exclusive of the adult phase).

**The authors only measure the expression in FPKM and then do, at least for me, an unusual selection of the top expressed genes (selection of top 50 in at least 4 out of 6?). From 100 top expressed genes in each library only 50 were common (sometimes not even in all replicates).**

Authors’ response: We apologize if the choice for showing the 50^th^ most expressed genes was not clear in the manuscript. The idea was to give to the reader a general idea about the genes with higher expression in the brain. The selection of this number is arbitrary, trying to avoid reporting a too extensive list. Therefore, we ranked library outputs according to gene FPKM values (now replaced by TPM), compared these ranks and built a consensus list depicting genes that ranked top 50 in at least four out of the six libraries. An additional criterion to complete this selection was that genes should present an annotation in VectorBase and/or a positive BLASTp output (against Insect class sequences in GenBanK) to grant informing their potential function. We have changed this information in the description of the results, that now reads:

“To characterize the set of genes most highly expressed in the brain, we compared these ranks and built a consensus list depicting genes that ranked top 50 in at least four out of six libraries”

**I think it would be nice to have a quality assessment of the datasets, showing for instance how different libraries compare to each other (PCA or some distance clustering).**

Authors’ response: Following the reviewer's suggestion, we added a PCA analysis in the new version of the manuscript. The S1 Fig includes a graph that summarizes the results of the PCA. Our results indicate that four libraries clustered together and two (one from each experiment) appeared segregated. To the best of our knowledge, we are satisfied with this result as it shows similar expression pattern throughout different experiments (see S1_table). Still, differences in gene expression where noticed and this could be related to the depth of the experiment itself.

**I believe that GO enrichment analysis should be performed. The way they do it throughout the paper is to get BLAST results from the selected genes and infer functions. GO analysis gives more statistical information about the different functions associated with a set of genes and probably should be done together with the BLAST analysis.**

Authors’ response: This is a very interesting suggestion. However, a proper GO enrichment analysis is based on the annotation of the GO-terms of a whole gene set. This process is currently undergoing work in *R. prolixus*. In fact, more than 30% of the *R. prolixus* genes has no single GO annotation (information from VectorBase). We consider that using such a limited background to identify GO enriched terms could lead to false conclusions regarding the R. prolixus gene dataset. Therefore, we would like to rely on our results based on BLAST hits as originally presented.

**The quality of all the figures is below publication standards. I had hard times reading numbers and letters in all the heatmaps.**

Authors’ response: We apologize for this. We have included new figures with the quality requested in the PLOS instructions for authors.

**Line 137 "Gene length of mapped reads". The sentence should probably be "Gene length and total counts of mapped reads were used"**

Authors’ response: The change was made as suggested.

**Line 138 The authors should use TPM instead of FPKM since FPKM (and RPKM) were already shown to be inconsistent across replicates (Wagner et al, 2012 - doi.org/10.1007/s12064-012-0162-3)**

Authors’ response: We appreciate the recommendation. Therefore, we have made new calculations to express all the values as TPM.

**Line 143 "The identity and putative functions" should be changed to "To identify putative functions"**

Authors’ response: The change was made as requested.

**Reviewer #2:**

**Major points: Materials and methods**

**The aim of this study was to focus on the starved stages of R. prolixus but no description on how the nymphs were prepared.**

Authors’ response: We apologize for the mistake. The following sentence was added to the text:

“For this study, seven day-old 4^th^ instar nymphs were fed on citrated rabbit blood using an artificial feeder. Insects were kept unfed for 30 days after their ecdysis to the 5^th^ instar and subsequently dissected to collect their brains.”

**Line 121-122: Six independent replicates was performed, however, it seems that no experiment designs were conducted, which made the results more descriptive but not problem-orientated**.

Authors’ response: We thank the reviewer for the interesting comments. Regarding the use of six libraries of unfed bugs in our work, we want to clarify that they belong to an originally larger set of samples where the initial objective was to compare gene expression profiles between the brains of healthy and Trypanosoma cruzi-infected nymphs (infected either with the CL or Dm28c *T. cruzi* strains, each with 3 biological replicates*)*. We decided not to include the comparison between infected and uninfected insects in this manuscript because the differential expression analyses showed no significant differences associated with parasite infection. *Trypanosoma cruzi* is a parasite that lives exclusively in the insect’s intestinal tract, and the changes in gene expression triggered in the *R. prolixus* brain may be subtle or restricted to few neurons in specific brain regions, being masked when the whole brain is analyzed. In fact, it has been shown that even the comparison between fed and unfed *R. prolixus* adults using RNA-Seq showed no significant changes in the expression of neuropeptide and neurohormonal signaling genes in the central nervous system (Leryia et al. 2020), reflecting the challenge of detecting gene expression differences in this tissue. Therefore, we decided to use the control samples (6 replicates) to describe the overall expression of genes in unfed nymphs because we felt that describing the expression of genes potentially involved in modulating behavior during starvation will contribute to a better understanding of the functional genomics of triatomines. Nevertheless, if the reviewer considers it relevant to include the data referring to *T. cruzi*, we can prepare a new version of our manuscript containing the brain libraries of *T. cruzi*-infected nymphs.

Leyria J, Orchard I, Lange AB. Transcriptomic analysis of regulatory pathways involved in female reproductive physiology of *Rhodnius prolixus* under different nutritional states. Sci Rep. 2020;10(1).

**The format of figures had a single form and the results seems to be descriptive and deductive.**

Authors’ response: The figures try to show, using different colors, the differences in the level of expression of genes belonging to several families. We have standardized them to allow the reader to have some degree of comparison.

**Minor points:**

**Line 79: R. prolixus shall be more suitable to provide a full name for the first time. In addition, please add some explanation on the relationship between R. prolixus and triatomines.**

Authors’ response: We apologize for the mistake. We included the full name of *R. prolixus*, as well as its biological classification.

**Line 443-664: The format of reference should be carefully checked.**

Authors’ response: The format of the references has been checked as requested.

**Table 2: Traces of revision was ignored.**

Authors’ response: We have checked and corrected the forgotten revision traces.
